# Comparative survival analysis of breast cancer microarray studies identifies important prognostic genetic pathways

**DOI:** 10.1186/1471-2407-10-573

**Published:** 2010-10-21

**Authors:** Jeffrey C Miecznikowski, Dan Wang, Song Liu, Lara Sucheston, David Gold

**Affiliations:** 1 Department of Biostatistics, University at Buffalo (SUNY), Buffalo, New York 14214 USA; 2 Department of Biostatistics, Roswell Park Cancer Institute, Buffalo, New York 14263 USA

## Abstract

**Background:**

An estimated 12% of females in the United States will develop breast cancer in their lifetime. Although, there are advances in treatment options including surgery and chemotherapy, breast cancer is still the second most lethal cancer in women. Thus, there is a clear need for better methods to predict prognosis for each breast cancer patient. With the advent of large genetic databases and the reduction in cost for the experiments, researchers are faced with choosing from a large pool of potential prognostic markers from numerous breast cancer gene expression profile studies.

**Methods:**

Five microarray datasets related to breast cancer were examined using gene set analysis and the cancers were categorized into different subtypes using a scoring system based on genetic pathway activity.

**Results:**

We have observed that significant genes in the individual studies show little reproducibility across the datasets. From our comparative analysis, using gene pathways with clinical variables is more reliable across studies and shows promise in assessing a patient's prognosis.

**Conclusions:**

This study concludes that, in light of clinical variables, there are significant gene pathways in common across the datasets. Specifically, several pathways can further significantly stratify patients for survival. These candidate pathways should help to develop a panel of significant biomarkers for the prognosis of breast cancer patients in a clinical setting.

## Background

Developing genomic based biomarkers for breast cancer prognosis is an active research area with clinicians and researchers considering genomic expression data as a potential valuable source of information to be mined for such markers. In addition to considering the BRCA mutation status of a patient, three genetic markers, estrogen receptors (ER) [1], progesterone receptors (PR) [2], and the HER2/neu receptor (HER2) [3] are commonly used for assessing prognosis and/or assigning treatment. More recently TGF- has also been considered as a potential prognosis biomarker [4].

One of the biggest challenges in developing valid prognostic genomic based biomarkers for breast cancer is obtaining large enough datasets with sufficient patient follow-up time [5,6]. To address this, we employ a comparative analysis approach. In a comparative analysis, several datasets gathered to test related hypotheses are combined to obtain more powerful estimates for a common hypothesis. We combine five genomic studies examining prognosis in breast cancer patients to assess the ability of the genetic biomarkers to stratify or distinguish patient survival. Datasets under consideration were chosen based on sample size and the availability of gene expression microarray data derived from RNA extracted from breast cancer tumors with sufficient follow-up data. At the time of this analysis, five datasets were publicly available; we reference these by primary author: Desmedt [7] (data accessible at NCBI GEO database [8], accession GSE7390), Miller [9] (accession GSE3494), Pawitan [10] (accession GSE1456), VAN DE Vijver [11]http://microarray-pubs.stanford.edu/wound_NKI/, and Bild [12] (accession GSE3143). While we find that that individual gene analysis results are highly variable across similar datasets, using a gene pathways analysis approach shows promising evidence that genetic pathways can further stratify survival across datasets.

## Methods

### Data Collection and Pre-processing

The breast cancer microarray datasets were either downloaded from the NCBI GEO database or provided by the authors through their public websites. Among the five datasets, three were based on the Affymetrix U133 platform, one on the Affymetrix U95 platform, and one using the Agilent two-color platform (Table 1). The four Affymetrix based datasets were processed using the RMA algorithm in the "affy" R library within the Bioconductor suite to generate expression summary values [13-15]. The expression summary values for the Agilent platform were directly taken from Chang et al.[16]. The NCBI entrez gene names were assigned to all of the Affymetrix probes and Agilent cDNA clones based on latest Bioconductor annotation package [13,17]. Note that only 12,649 Agilent cDNA clones were successfully mapped to the latest entrez gene annotation used in our analysis. We obtained the patient specific clinical data through the primary author's public website or via communication with the authors. The clinical demographics for each of the datasets is provided in the Additional File 1.

**Table 1:** Microarray Dataset Summary

Dataset	Total Samples	Array Description	Total Probes	Years of Diagnosis
Desmedt (GSE7390)	198	Affymetrix U133A	22283	1980-1998

Miller (GSE3494)	251	Affymetrix U133A	22283	1987-1989

Pawitan (GSE1456)	159	Affymetrix U133	22283	1994-1996

VAN DE Vijver	295	Agilent	24481*	1984-1995

Bild (GSE3143)	158	Affymetrix Hu95Av2	12625	-

* only 12649 probes were used for analysis

### Survival Analysis

The Cox proportional hazards regression model was used to discover significant variables correlated with risk with reported p-values obtained from a Wald test [18]. Overall survival was used as the endpoint in each analysis, except in the Miller dataset where disease specific survival was the only available endpoint. Both univariate and multivariate survival analysis were performed to select the clinical variables and/or their interactions significant in each of the datasets. Model fitting for each gene expression profile was determined by using each gene 1) individually, 2) in conjunction with ER status and tumor size, 3) with the best model from minimizing Akaike's information criterion (AIC), and 4) minimizing the Bayesian information criterion (BIC) [19]. The summary for each AIC and BIC based model using only the clinical variables in each dataset is shown in Table 2. For each gene model, the statistical significance for individual genes was determined by controlling the false discovery rate (FDR) for testing multiple genes at 0.2 using a Benjamini and Hochberg scheme for the p-values obtained from log-rank tests [20].

**Table 2:** AIC and BIC Model Summary

	Datasets				
**Variables**	**Desmedt**	**Miller**	**Pawitan**	**VAN DE Vijver**	**Bild**

ER status	*√ *†			*√ *†	*√ *†

tumor size	*√*	*√ *†	n.a.	*√ *†	*√ *†

tumor grade				*√*	n.a.

patient age	*√*			*√*	n.a.

lymph status	n.a.	*√ *†	n.a.	n.a.	n.a.

number positive lymph (NPL)	n.a.	n.a.	n.a.	*√ *†	n.a.

p53 status	n.a.	*√*	n.a.	n.a.	n.a.

x70 status [34]	n.a.	n.a.	n.a.	*√ *†	n.a.

Surgery type	*√*	n.a.	n.a.	n.a.	n.a.

subtype	n.a.	n.a.	√	n.a.	n.a.

patient age*surgery type	*√*	n.a.	n.a.	n.a.	n.a.

patient age*grade	n.a.	n.a.	n.a.	*√*	n.a.

x70* NPL	n.a.	n.a.	n.a.	*√*	n.a.

x70*tumor grade	n.a.	n.a.	n.a.	*√*	n.a.

Note: *√ *= variable is significant in AIC model, † = variable is significant in BIC model, n.a.= variable not available

### Pathway Analysis

The pathway database was compiled from the Kyoto Encyclopedia of Genes and Genomes (KEGG) [21] with the addition of curated pathways from the human protein reference database (HPRD) [22]. The combined KEGG and HPRD pathway database contains 232 human pathways that include metabolism, genetic information processing, environmental information processing, cellular processes, human diseases, and drug development. Note, for the sake of interpretation, 175 pathways passed our gene pathway size filter criteria (min = 15 probes; max = 250 probes). Since the development of the gene set enrichment analysis (GSEA) algorithm [23], researchers have been able to use gene pathways (sets) to capture molecular dysregulation even when individual genes are highly variable. A modified Gene Set Analysis (GSA) method was used to measure gene correlation with overall survival after accounting for ER status and tumor size [24]. GSA offers two potential improvements to GSEA, namely the maxmean statistic for summarizing gene sets and restandardization for more accurate inferences. The restandardization process consists of a randomization step and a permutation step. The randomization step standardizes the maxmean statistic with respect to its randomized mean and standard deviation then the permutation step computes the p-value for the statistic from a permutation distribution.

The generalized Šidàk-Holm method was used to determine the significance of a given pathway, where the generalized family wise error rate (gFWER) was controlled at 0.20 with the number of false discoveries limited to five [25]. In short, our gFWER procedure controls the probability of committing five or more false positives to be no larger than 0.20. To further explore and graphically display the large number of significant gene pathways associated with the survival results from GSA, we devised a voting mechanism to stratify subjects by pathway activity. In this way, we can explore the contribution of each gene in a significant gene pathway. We define the pathway risk index (PRI) for subject *j *and pathway *k *as

$$\text{PRI}(j,k)=\sum_{i=1}^{G_{k}}I(x_{ij}>\bar{x_{i}})$$

where *I *() denotes the indicator function, *x_ij _*is the expression value of the *i*th gene on subject *j*, $\bar{x_{i}}$ is the sample mean for the *i*th gene, and *G_k _*is the number of genes in pathway *k*. For each pathway, the PRI score for a subject is further stratified into low PRI (below median PRI) or high PRI (above median PRI). Low PRI indicates that many of the genes in pathway *k *for subject *j *tend to be expressed below their mean expression, while high PRI for a subject indicates that many genes in pathway *k *tend to be expressed above their mean expression. The PRI is well suited for molecular stratification when combined with the results of GSA. By using the modified version of GSA, we stratify study populations according to both clinical and molecular covariates. Thus, the PRI score for a pathway provides the marginal benefit of that pathway to explain survival in light of tumor size and ER status. Note that PRI does not account for mixed direction of gene expression change in a gene set, but rather is a global signature designed to summarize the gene activity within a pathway across the set of patients. We then used the PRI score to further stratify survival within a subset of patients.

### Comparative Analysis

**Figure 1:**
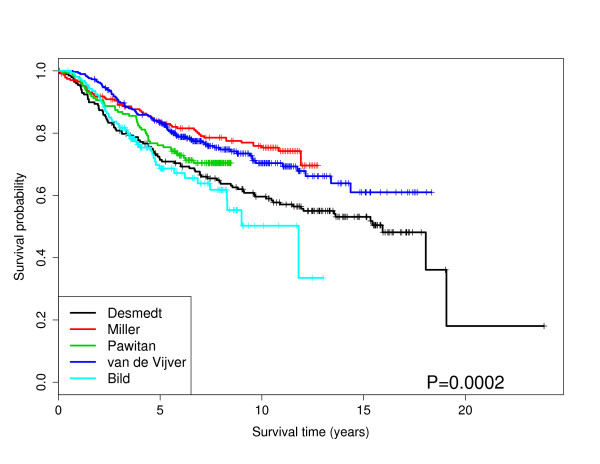
**Kaplan-Meier Curves**: The survival curves for each dataset. The p-value is from a Wald test. The survival probabilities are obtained from Kaplan-Meier estimates.

**Figure 2:**
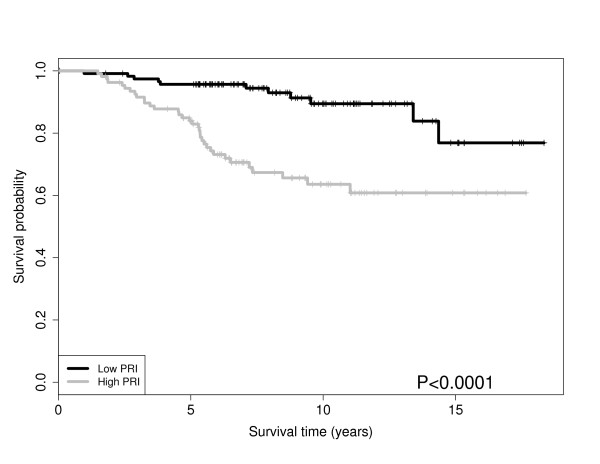
**Pathway PRI Stratifies Grade and Survival**: Survival for the ER positive patients stratified by PRI score for *cell cycle *pathway in VAN DE Vijver. The p-value is from a Wald test. The survival probabilities are obtained from Kaplan-Meier estimates.

Our goal was to discover shared genes and/or pathways that represent pseudo-global biological and molecular mechanisms associated with breast cancer survival while accounting for clinical covariates that can explain inter-study dissemblance, that is, known clinical predictors of clinical outcome. To this end we compared the results from the gene and gene pathway inference across datasets for each analysis. The results are displayed in the graphical and tabular summaries (Tables 1,2,3,4 and Figures 1,2) including Venn diagrams in Additional File 1. For significantly enriched pathways in more than one study, we perform multivariate analysis on pathway gene expression between datasets using the PRI scores to learn of pseudo-types. In other words, for enriched pathways, we examine the pathway signature within patient cohorts, such as the cohort of ER positive patients and the cohort of patients with the same tumor grade (see Pathway Analysis section).

**Table 3:** Number of Significant Genes

	Desmedt	Miller	Pawitan	VAN DE Vijver	Bild
Univariate	5	1886	1404	3246	138

ER status + tumor size	3	534	1487	483	6

AIC Best Model	3	31	2	22	6

BIC Best Model	1	123	1404	35	6

**Table 4:** Comparative Analysis Results:

Pathway (# of probes)	Desmedt	Miller	Pawitan	VAN DE Vijver	Bild
*pyrimidine metabolism *(77)		*√*	*√*	*√*	

*Carbon fixation *(21)		*√*	*√*		*√*

*biosynthesis of phenylpropanoids *(31)		*√*	*√*	*√*	*√*

*DNA replication *(34)		*√*	*√*	*√*	

*cell cycle *(104)		*√*	*√*	*√*	*√*

*IL-7 Signaling *(16)		*√*	*√*	*√*	

*bladder cancer *(39)	*√*		*√*	*√*	

Note: √ = pathway is significant

## Results

Exploratory graphical analyses of the clinical covariates with survival are available in our Additional File 1. Treatment regimens and survival distributions are known to differ by dataset. Figure 1 shows the Kaplan-Meier curves for each dataset. Note, all datasets included tumor size and ER status with the exception of Pawitan. We modeled the overall survival using the gene expression microarray datasets with a series of Cox proportional hazards models. It is noteworthy that tumor size was significant (when available) for all datasets while ER status was highly significant (p-value < 0.01) in three out of five datasets. Table 2 displays the significant variables for the AIC and BIC models using only the clinical variables as described in the Materials and Methods section. From Table 2, the AIC models tend to yield larger models than the BIC models while tumor size and ER status are significant in most cases.

Table 3 shows the results from the survival analysis using the gene expression data with the four models discussed in Material and Methods. Table 3 shows that the Miller, Pawitan and VAN DE Vijver have a large number of discoveries in the univariate gene models and the gene models including ER status and tumor size, but very few discoveries in the AIC models. Also, the Desmedt dataset shows very few discoveries regardless of the model. Ultimately, the molecular variability of the genes within these pathways tended to be discordant, that is, the genes with the strongest correlation with risk were miscellaneous.

**Figure 3:**
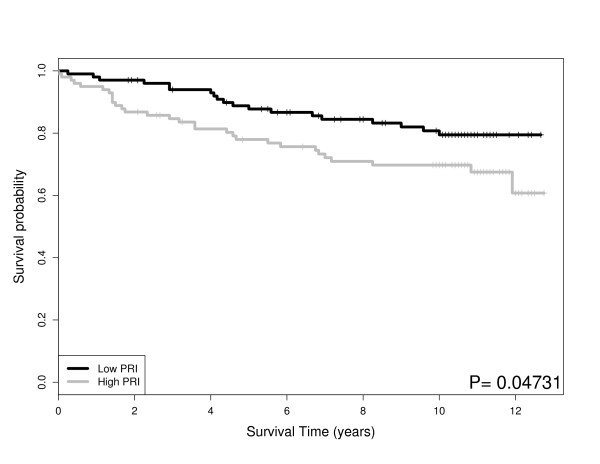
**Pathway PRI Stratifies Grade and Survival**: Survival for the ER positive patients stratified by PRI score for *cell cycle *pathway in the Miller dataset. The p-value is from a Wald test. The survival probabilities are obtained from Kaplan-Meier estimates.

**Figure 4:**
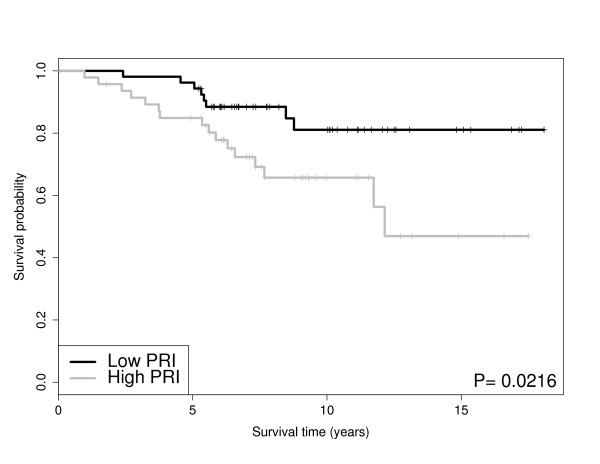
**Pathway PRI Stratifies Grade and Survival**: PRI score for *biosynthesis of phenylpropanoids *pathway for intermediate tumor grade patients in VAN DE Vijver dataset. The p-value is from a Wald test. The survival probabilities are obtained from Kaplan-Meier estimates.

The gene pathway analysis results are displayed in Table 4. For the four datasets including ER status and tumor size (Desmedt, Miller, VAN DE Vijver, Bild), we performed pathway analysis, accounting for ER status and tumor size. Table 4 shows that the *biosynthesis of phenylpropanoids *pathway and *cell cycle *pathway were discovered in four data sets. Other pathways found in three of the datasets include the pyrimidine *metabolism, tAminoacyl-tRNA biosynthesis, DNA replication, IL-7 Signaling and bladder cancer *pathways. Accounting for ER status and tumor size dramatically reduced the number of significant pathways present in at least three datasets. Figure 2,3,4, and 5 shows the Kaplan-Meier curves stratified by the PRI scores (Low vs. High) for two of the selected pathways in Table 4; the *cell cycle *pathway and the *biosynthesis of phenylpropanoids *pathway.

**Figure 5:**
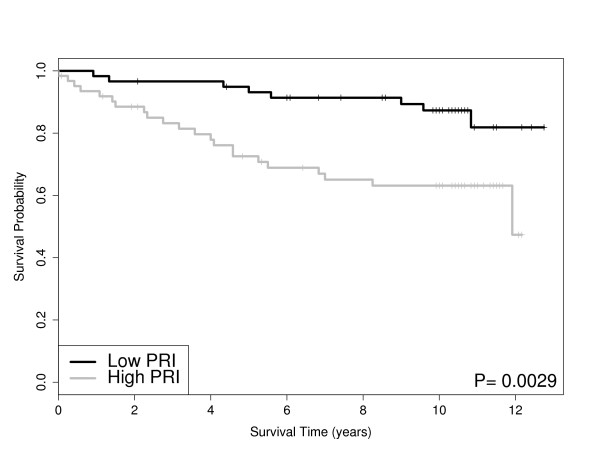
**Pathway PRI Stratifies Grade and Survival**: PRI score for *biosynthesis of phenylpropanoids *pathway for the tumor grade three patients in Miller dataset. The p-value is from a Wald test. The survival probabilities are obtained from Kaplan-Meier estimates.

The *cell cycle *pathway for ER positive patients significantly stratifies survival in the VAN DE Vijver dataset and the Miller dataset as shown in Figures 2 and 3, respectively. In other words, the *cell cycle *pathway stratification appears to explain additional variation beyond ER status alone. We found these results encouraging, as the *cell cycle *pathway is known to be disrupted in general cancers [26] and Specifically breast cancer [27,28]. Note, that similar results for patient survival apply to the *pyrimidine metabolism *pathway which is known to be connected to energy metabolism, cell growth and proliferation and is an active pathway in human leukocytes [29]. In addition to the cohort of ER positive patients, the PRI scores also stratify survival within a cohort of patients with the same tumor grade. Figures 4 and 5 show the *biosynthesis of phenylpropanoids *pathway can significantly stratify survival in a cohort of intermediate tumor grade patients in the VAN DE Vijver dataset and a cohort of tumor grade three patients in the Miller dataset, respectively.

The differentiation and proliferation of some haematological malignancies is known to be induced by Interleukin-7 (IL-7), a haematopoietic growth factor. While not much is known about its role in solid tumors, recently it was shown that aberrant expression of IL-7 and its signaling intermediates in invasive breast cancers could have significant diagnostic and prognostic implications. Thus measuring these molecules in breast cancer tissues may provide important molecular indicators of tumor differentiation, aggressiveness, nodal status, prognosis and patient survival [30].

## Discussion

With the increasing availability of genome wide data, comparative meta-analyses offer researchers an exciting opportunity to obtain generalizable results with appropriate statistical power. There are several examples of meta-analyses and re-analysis of publicly available datasets related to breast cancer research [31-33]. However, there are challenges to consider when performing a meta-analysis, including inter-study differences, lack of variables in common, significant sample size differences, and the inability to validate results across datasets. These concerns are especially relevant in cancer datasets where there can be large differences in results due to the genetics, race, epidemiology, treatment, and age differences in the patient cohorts. Further the nature of microarray based datasets can suffer from lab specific variability, probe variability, chip to chip variation and sample preparation(s) required for each experiment. These individual breast cancer studies each have their own complexities and the inter-study differences are well documented.

Ultimately, sample size, distinct study disease populations, and departures in treatment regimens preclude directly combining data, or pooling analyses, for the sake of meaningful prediction. For example, the Miller dataset has the highest mean patient age (see Additional File 1) and the longest mean patient survival times (see Figure 1). This evidence suggests that predominantly post-menopausal women (most likely with sporadic disease) comprise the Miller dataset. Taken in conjunction with the low proportion of women with ER negative tumors (< 25%), one might not expect as prominent a genetic signature. This may explain at least in part the ability of PRI to stratify subject survival in grade three cancers (see Table 3 and Figure 5). Attention to these details are important for correct interpretation of our comparative analysis results. To overcome some of these challenges for our comparative analysis, we examined the marginal utility of well accepted clinical cancer biomarkers, such as ER status and tumor size as measured via computed tomography (CT) and magnetic resonance (MR) imaging. We recognize that tumor grade may also have utility in predicting patient prognosis, however, we did not consider tumor grade within our class of models due to the potential subjectivity of pathology scores and the potential confounding with tumor size. Further, tumor grade was not available for all of our datasets and by using tumor size in our models, we believe we have an adequate surrogate for tumor grade. Unfortunately TNM (tumor-node-metastasis) classification is not available for all of our datasets, however, we do use tumor size in our models which forms part of the TNM classification system. Besides ER status, other documented genetic variables important in breast cancer research include progesterone receptor status (PR), the HER2/neu receptor, and the BRCA1 and BRCA2 mutation status. Unfortunately, these variables were not consistently available across all of the datasets, hence we were unable to study their utility in assessing survival across each of the datasets. Ultimately, the results from a gene pathway analysis usually consist of a list of genetic pathways that are significantly associated with prognosis. However, this list of pathways is of little practical use for clinicians. That is, a list of significant pathways does not directly help a patient or an oncologist in choosing an optimal treatment plan. However, these lists of significant pathways are important at a systems biology level in aiding future exploration of drug targets and their effects on critical nodes in specific pathways. To further extend the gene pathway analysis results, we develop a scoring metric called the pathway risk indicator (PRI) to summarize the results for a given pathway. By using the PRI to summarize the gene pathway signature for each patient to a scalar score, we are creating a robust measure of that pathway's ability to explain survival. This method to reduce variability shows large reproducibility across datasets and offer clinicians a chance to offer better treatment options for their patients. The nature of the PRI score for a given pathway allows for the following interpretation. A high PRI score indicates that the patient has a large number of gene expression values in given pathway higher than the mean expression value across all patients. A low PRI score implies the patient had a smaller than average score for each gene in that pathway. Thus the PRI score, in a sense, measures the activity of the pathway for that patient. For the significant pathways associated with survival as determined by GSA (*e.g. cell cycle and pyrimidine metabolism pathways*), we find that the PRI score for these pathways can further stratify patients after controlling for ER status and tumor size. For further research our group is examining other potential metrics (equations) for PRI scores.

## Conclusions

The comparative analysis on cancer datasets offers researchers an opportunity to gain statistical power in researching genetic biomarkers for cancer and the opportunity to generalize the results to a larger population. Previous studies for cancer prognosis have tended to focus either on the molecular or genetic characteristics for the patients or solely on the clinical characteristics for the patients. This comparative analysis combines the clinical and molecular data for each patient to determine the optimal set of variables that explain survival in each dataset. Ultimately, ER status and tumor size were the most significant molecular variables. In the molecular analysis, we have combined five microarray datasets to examine the ability of genetic biomarkers to stratify survival for breast cancer patients. Using a series of Cox proportional hazards models, there is little overlap in the sets of significant genes associated with survival in each dataset. However, when extending the survival analysis to include gene pathway analysis, there are several genetic pathways that are significant in a number of the datasets. Using the pathway risk index (PRI), we show that cohorts of patients, Specifically ER positive patients and patients with the same tumor grade, can be stratified for survival even when considering the clinical variables of ER status and tumor size. Specifically, the pathways in Table 4 have the most significance across the five datasets for stratifying survival using the PRI. Ultimately, this analysis combines aspects of a patients clinical profile with their molecular profile and allows clinicians the opportunity to further stratify survival following surgery and chemotherapy in breast cancer patients.

## Competing interests

The authors declare that they have no competing interests.

## Authors' contributions

JCM designed the analysis, analyzed the data, and contributed to the writing of the manuscript. DW performed the data analysis, contributed to the writing of the manuscript, and provided the figures and tables. SL designed the analysis, analyzed the data, and contributed to the writing of the manuscript. LS analyzed the data and contributed to the writing of the manuscript. DG designed the analysis, analyzed the data, and contributed to the writing of the manuscript. All authors read and approved the final manuscript.

## Pre-publication history

The pre-publication history for this paper can be accessed here:

http://www.biomedcentral.com/1471-2407/10/573/prepub

## Supplementary Material

Additional file 1 **Additional Materials**. metapaper.supp.pdf - An additional file (PDF) showing additional tables and results. Click here for file
