# Supplementary material for: Comparative survival analysis of breast cancer microarray studies identifies important prognostic genetic pathways
Source: BMC Cancer. 2010 Oct 21;10:573. doi: 10.1186/1471-2407-10-573 (PMC2972286; doi:10.1186/1471-2407-10-573)
Supplement: Additional file 1 — Additional Materials. metapaper.supp.pdf - An additional file (PDF) showing additional tables and results. [file 1471-2407-10-573-S1.PDF]

Additional materials: comparative survival analysis of breast cancer microarray studies identifies important prognostic genetic pathways

Jeffrey C. Miecznikowski*†, Dan Wang†,
Song Liu†, Lara Sucheston†, and David Gold†

*Corresponding Author: Email: jcm38@buffalo.edu, Phone: (716) 881.8953, Fax (716) 829.2200

†Department of Biostatistics; University of Buffalo; Buffalo, NY 14214-3000 USA Department of Biostatistics; Roswell Park Cancer Institute; Buffalo, NY 14263 USA.

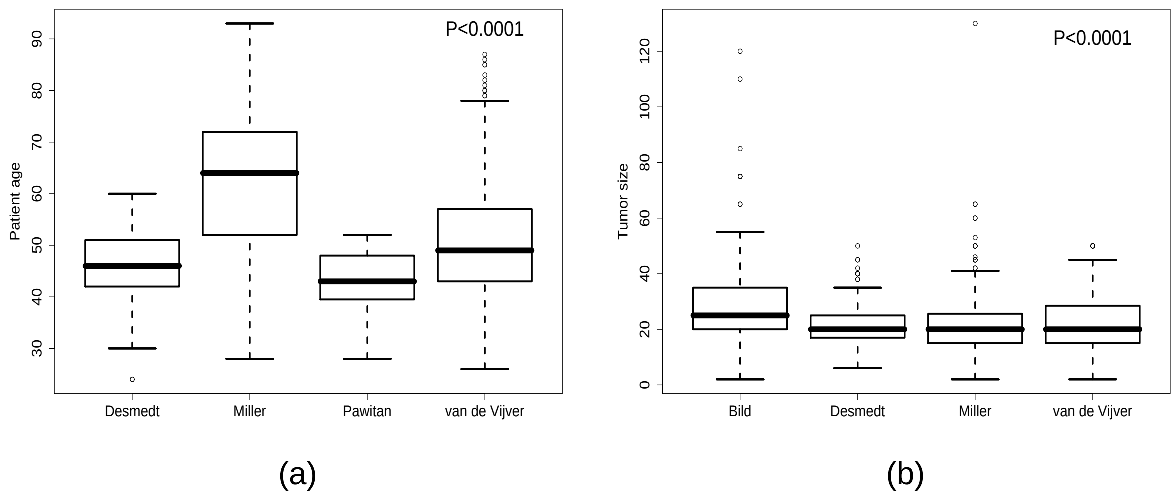

Figure S 1: **Patient Age and Tumor Size:** (a) Patient age and (b) tumor size comparison across datasets. The p-values are calculated from an F test.

Characteristic	**Poor-Prognosis**	**Good-Prognosis**
	N=32	N=164
	no.of patients(%)	
Age		
<40 yr	10(5)	25(13)
40-44 yr	8(4)	36(18)
45-49 yr	8(4)	43(22)
$\geq$ 50 yr	6(3)	60(31)
Histologic Grade		
I(good)	4(2)	26(13)
II(intermediate)	9(5)	74(38)
III(poor)	19(10)	64(33)
ER status		
ER-	19(10)	45(23)
ER+	13(7)	119(61)
Tumor diameter		
$\leq$20 mm	4(2)	63(32)
>20 mm	28(14)	101(52)
Surgtype		
0	22(11)	119(61)
1	10(5)	45(23)

Table S 1: **Clinical Characteristics-Desmedt:** A table containing the Association Between Clinical Characteristics and the Prognosis Signature.

Characteristic	**Poor-Prognosis**	**Good-Prognosis**
	N=59	N=177
	no.of patients(%)	
Age		
<40 yr	2(1)	13(6)
40-44 yr	1(0)	11(5)
45-49 yr	4(2)	15(6)
≥ 50 yr	52(22)	138(58)
Histologic Grade		
I(good)	6(3)	56(24)
II(intermediate)	27(11)	94(40)
III(poor)	25(11)	26(11)
ER status		
ER+	48(20)	153(65)
ER-	10(4)	21(9)
ER?	1(0)	3(1)
Tumor diameter		
≤20 mm	14(6)	91(39)
>20 mm	45(19)	86(36)
p53 Status		
p53+	23(10)	32(14)
p53-	36(15)	145(61)
Lymph node status		
LN+	35(15)	43(18)
LN-	16(7)	133(56)
LN?	8(3)	1(0)

Table S 2: **Clinical Characteristics-Miller:** A table containing the association between clinical characteristics and the prognosis signature.

Characteristic	**Poor-Prognosis**	**Good-Prognosis**
	N=63	N=232
	no.of patients(%)	
Age		
<40 yr	24(8)	39(13)
40-44 yr	13(4)	72(24)
45-49 yr	16(5)	82(28)
$\geq$ 50 yr	10(3)	39(13)
Histologic Grade		
I(good)	5(2)	70(24)
II(intermediate)	46(16)	73(25)
III(poor)	12(4)	89(30)
ER status		
ER	30(10)	39(13)
ER+	33(11)	193(65)
Tumor diameter		
$\leq$20 mm	16(5)	98(33)
>20 mm	47(16)	134(45)
Hormonal therapy		
Yes	7(2)	33(11)
No	59(19)	199(67)
No. of positive nodes		
0	35(12)	116(39)
1-3	15(5)	91(31)
$\geq$4	14(5)	10(3)
Chemotherapy		
Yes	16(5)	94(32)
No	47(16)	138(47)

Table S 3: **Clinical Characteristics- VAN DE Vijver:** A table containing the association between clinical characteristics and the prognosis signature.

Characteristic	**Poor-Prognosis**	**Good-Prognosis**
	N=34	N=110
	no.of patients(%)	
Age		
<40 yr	7(5)	7(5)
40-44 yr	2(1)	8(6)
45-49 yr	4(3)	17(12)
≥ 50 yr	21(15)	78(54)
Histologic Grade		
I(good)	3(2)	24(17)
II(intermediate)	13(9)	45(31)
III(poor)	18(12)	41(28)
ER status		
ER+	28(19)	91(63)
ER-	6(4)	19(13)
Subtype		
Basal	6(4)	17(12)
ERBB2	5(3)	8(6)
Luminal A	8(6)	26(17)
Luminal B	10(7)	12(8)
Normal Like	2(1)	32(22)
No Subtype	3(2)	16(11)
Hormonal Therapy		
Former	0(0)	8(6)
Never	29(20)	72(50)
Ongoing	5(3)	30(21)

Table S 4: **Clinical Characteristics-Pawitan:** A table containing the association between clinical characteristics and the prognosis signature.

Characteristic	**Poor-Prognosis**	**Good-Prognosis**
	N=98	N=60
	no.of patients(%)	
ER status		
ER-	35(22)	13(8)
ER+	63(40)	47(30)
Tumor diameter		
≤20 mm	19(12)	20(13)
>20 mm	79(50)	40(25)

Table S 5: **Clinical Characteristics-Bild:** A table containing the association between clinical characteristics and the prognosis signature.

Variable	Desmedt	Miller	Pawitan	VAN DE Vijver	Bild
patient age	0.479	0.788	0.207	**0.005**	—
ER Status	**0.007**	0.346	0.864	$<$**0.001**	**0.006**
tumor size	**0.019**	$<$**0.001**	—	**0.002**	**0.015**
Grade	0.643	**0.003**	0.292	$<$**0.001**	—
Menopause Status	0.967	0.962	0.143	0.259	—

Table S 6: **Univariate P-values:** A table containing the univariate (unadjusted) p-values for the clinical variables with overall survival as determined by a log rank test for each dataset.

Variables	Desmedt	Miller	Pawitan	VAN DE Vijver	Bild
ER status	$\surd$	$\surd$	$\surd$	$\surd$	$\surd$
Tumor size	$\surd$	$\surd$		$\surd$	$\surd$
Tumor grade	$\surd$	$\surd$	$\surd$	$\surd$	
Patient age	$\surd$	$\surd$	$\surd$	$\surd$	
Menopause Status	$\surd$	$\surd$	$\surd$	$\surd$	
Lymph status		$\surd$			
Number of positive lymph (NPL)				$\surd$	
p53 status		$\surd$			
Surgery type	$\surd$				
Disease subtype			$\surd$		
Hormonal receptor status			$\surd$		

Table S 7: **Clinical Variables:** A table containing the available clinical variables for each data set.

	Desmedt	Miller	Pawitan	VAN DE Vijver	Bild
ER +	0.677	0.849	0.818	0.766	0.696
ER -	0.323	0.136	0.182	0.234	0.304
ER ?	0	0.015	0	0	0

Table S 8: **ER Status Summary:** The summary results showing the percentage of patients in each study separated according to estrogen receptor positive (ER +), estrogen receptor negative (ER -) and estrogen receptor status unknown (ER ?).

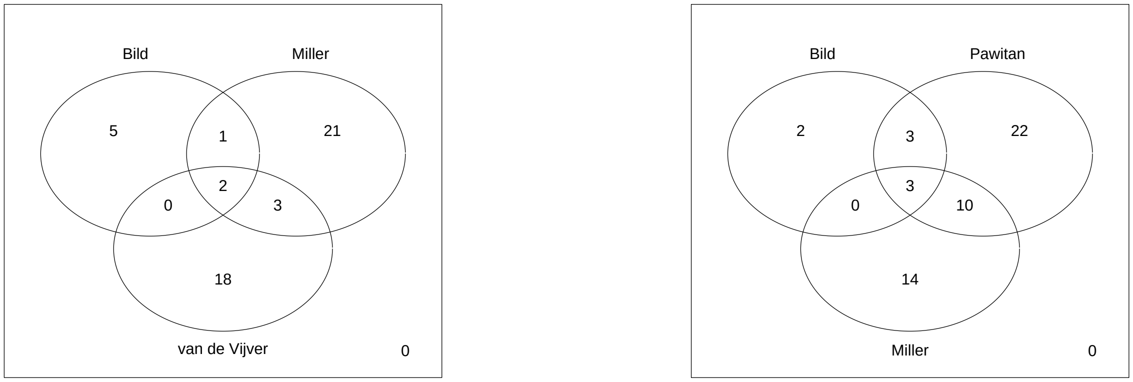

(A) (B)

Figure S 2: **Venn Diagram for Gene Pathways:** Venn Diagram shows the number of significant gene pathways across the datasets in models containing ER status and tumor size. Significance was determined by controlling gFWER such that the probability of committing five false positives is no more than 0.20 via the generalized Šidàk-Holm procedure [1]. (A) The Venn diagram for the Bild, Vijver, and Miller datasets. (B) The Venn diagram for the Bild, Pawitan, and Miller datasets.

Datasets	Covariates	coef	exp(coef)	se(coef)	z	p
Desmedt AIC	ER status	-0.753	0.471	0.286	-2.64	0.008
	tumor size	0.266	1.305	0.154	1.72	0.085
	age	-0.026	0.975	0.025	-1.04	0.300
	Surgtype	-5.532	0.004	2.075	-2.67	0.008
	age:Surgtype	0.115	1.121	0.042	2.74	0.006
Desmedt BIC	ER status	-0.722	0.486	0.269	-2.68	0.007
Miller AIC	tumor size	0.032	1.030	0.012	2.71	0.007
	p53 status	0.463	1.590	0.300	1.54	0.120
	Lymph status	1.135	3.110	0.295	3.85	0.0001
Miller BIC	tumor size	0.036	1.040	0.011	3.210	0.001
	Lymph status	1.129	3.090	0.292	3.86	0.0001
VAN DE Vijver AIC	ER status	-0.492	0.612	0.250	-1.97	0.049
	tumor size	0.023	1.020	0.013	1.74	0.082
	patient age	-0.054	0.948	0.037	-1.43	0.150
	Grade2 vs Grade1	2.543	12.700	2.379	1.07	0.290
	Grade3 vs Grade1	-24.369	$<$0.001	11.445	-2.13	0.033
	X70[2]	4.156	63.800	1.362	3.05	0.002
	NPL	0.385	1.470	0.134	2.88	0.004
	patient age:Grade2	0.009	1.010	0.045	0.21	0.830
	patient age:Grade3	0.535	1.710	0.237	2.26	0.024
	X70 vs NPL	-0.369	0.691	0.143	-2.58	0.010
	Grade2:X70	-2.942	0.053	1.294	-2.28	0.023
	Grade3:X70	-2.294	0.101	1.559	-1.47	0.140
VAN DE Vijver BIC	tumor size	0.037	1.040	0.013	2.90	3.7e-03
	X70	2.128	8.400	0.397	5.36	8.1e-08
Pawitan AIC	Age	-0.0308	0.970	0.0211	-1.46	0.1400
	Hrt Never:Formor	-3.0755	0.0462	1.1335	-2.71	0.0067
	Hrt Ongoing:Formor	-2.6214	0.0727	1.1659	-2.25	0.0250
	Elston 2:1	-7.6294	4.86e-04	2.3193	-3.29	0.0010
	Elston 3:1	-4.6288	9.77e-03	2.0958	-2.21	0.0270
	Hrt Never:Elston 2	3.3733	0.292	1.3105	2.57	0.0100
	Hrt Ongoing:Elston 2	1.9297	6.89	1.3392	1.44	0.1500
	Hrt Never:Elston 3	2.9475	19.1	1.3401	2.20	0.0280
	Hrt Ongoing:Elston 3	3.4309	30.9	1.4422	2.38	0.0170
	Age:Elston 2	0.0784	1.08	0.0279	2.81	0.0050
	Age:Elston 3	0.0382	1.04	0.0248	1.54	0.1200
Pawitan BIC	—	—	—	—	—	—
Bild AIC/BIC	ER status	-0.782	0.457	0.288	-2.72	0.007
	tumor size	0.173	1.188	0.066	2.61	0.009

Table S 9: **Summary AIC and BIC Models:** The summary tables for each dataset showing the selected variables under the AIC based best model, BIC based best model. The table contains the estimated coeffi cient from a Cox Proportional Hazard model (coef), the exponentiated coeffi cient (exp(coef)), the standard error for the coeffi cient (se(coef)), the z-score (z) and the univariate p-value for association (p) based on the z-score.

References

1. Guo W, Romano J: **A generalized Sidak-Holm procedure and control of generalized error rates under independence**. *Statistical Applications in Genetics and Molecular Biology* 2007, **6**.

2. Van't Veer L, Dai H, van de Vijver M, He Y, Hart A, Mao M, Peterse H, Van der Kooy K, Marton M, Witteveen A, Schreiber G, Kerkhoven R, Roberts C, Linsley P, Bernards R, Friend S: **Gene expression profiling predicts clinical outcome of breast cancer.** *Nature* 2002, **415**(6871):530.
